# Prediction of survival in patients with advanced, refractory colorectal cancer in treatment with trifluridine/tipiracil: real-world vs clinical trial data

**DOI:** 10.1038/s41598-021-93732-5

**Published:** 2021-07-12

**Authors:** Ana Fernández Montes, Alberto Carmona-Bayonas, Paula Jimenez-Fonseca, Francisca Vázquez Rivera, Nieves Martinez Lago, Marta Covela Rúa, Antía Cousillas Castiñeiras, Paula Gonzalez Villarroel, Juan De la Cámara Gómez, José Carlos Méndez Méndez, Carmen Carriles Fernández, Manuel Sanchez Cánovas, Teresa Garcia García

**Affiliations:** 1 Medical Oncology Department, Complexo Hospitalario Universitario de Ourense, Ourense, Spain; 2 Department of Hematology and Medical Oncology, Hospital Universitario Morales Meseguer, UMU, IMIB, Murcia, Spain; 3 Department of Medical Oncology, Hospital Universitario Central of Asturias, IPSA, Oviedo, Spain; 4 Medical Oncology Department, Hospital Clínico Universitario de Santiago, Santiago, Spain; 5 Medical Oncology Department, Complexo Hospitalario Universitario A Coruña, A Coruña, Spain; 6 Medical Oncology Department, Hospital Universitario Lucus Augusti, Lugo, Spain; 7 Medical Oncology Department, Hospital Provincial de Pontevedra, Pontevedra, Spain; 8 Medical Oncology Department, Hospital Universitario Álvaro Cunqueiro, Vigo, Spain; 9 Medical Oncology Department, Hospital Universitario Arquitecto Marcide, Ferrol, Spain; 10 Medical Oncology Department, Centro Oncológico de Galicia, A Coruña, Spain; 11 Department of Pharmacy, Hospital Universitario Central of Asturias, IPSA, Oviedo, Spain; 12 Department of Hematology and Medical Oncology, Hospital Universitario Morales Meseguer, Murcia, Spain; 13 Department of Medical Oncology, Hospital Universitario Santa Lucía, Cartagena, Spain

**Keywords:** Medical research, Oncology

## Abstract

Trifluridine/tipiracil increases overall survival (OS) in patients with refractory, metastatic colorectal cancer (mCRC). A post hoc exploratory analysis of the RECOURSE randomized clinical trial (RCT) established two categories, a good prognosis corresponding to subjects having a low tumor burden and indolent disease. Other models in refractory mCRC are the FAS-CORRECT and Colon Life nomogram. The main objective was to externally validate the prognostic factors of the RECOURSE and FAS-CORRECT trials, and the Colon Life nomogram in a multicenter, real-world series of mCRC treated in 3rd and successive lines with trifluridine/tipiracil. The secondary aim was to develop an OS predictive model, TAS-RECOSMO. Between 2016 and 2019, 244 patients were recruited. Median OS was 8.15 vs 8.12 months for the poor (85% of the subjects) and good (15%) prognosis groups from the RESOURCE trial, respectively, log-rank p = 0.9. The most common grade 3–4 toxicities were neutropenia (17%), asthenia (6%), and anemia (5%). The AFT lognormal model TAS-RECOSMO included six variables: ECOG-PS, KRAS/NRAS/BRAF mutation status, time between diagnosis of metastasis and beginning of trifluridine/tipiracil, NLR, CEA, and alkaline phosphatase. The model’s bootstrapped bias-corrected c-index was 0.682 (95% CI, 0.636–0.722). The factors from the Colon Life model, FAS-CORRECT, and RECOURSE displayed a c-index of 0.690, 0.630, and 0.507, respectively. TAS-RECOSMO, FAS-CORRECT, and the Colon Life nomogram appear to predict OS in patients with refractory mCCR who begin trifluridine/tipiracil treatment in the real world. The prognostic groups of the RECOURCE RCT were unable to capture the situation of real-world subjects treated with trifluridine/tipiracil in this series.

## Introduction

Despite the inroads in targeted therapies made in recent years, individuals with refractory metastatic colorectal cancer (mCRC) have poor long-term survival. After progressing to second or third lines of chemotherapy, some patients maintain an acceptable functional situation and are eligible for successive therapies that prolong overall survival (OS) and preserve quality of life^1^. The RECOURSE randomized clinical trial (RCT) (NCT01607957) revealed that trifluridine/tipiracil increased OS vs placebo (median of 7.1 vs 5.3 months; hazard ratio [HR] 0.68, 95% confidence interval [CI], 0.58–0.81; *p* < 0.001) in individuals with refractory mCRC^2^. Trifluridine/tipiracil was effective in all subgroups^3^. Nevertheless, the OS benefit does not project equally to all groups, casting doubt on the use of aggressive therapies in subjects with an expectation of limited survival or at the end of life when the foreseeable benefit is diminished^4^.

Following this line of reasoning, Tabernero et al. evaluated the prognostic factors in the RECOURSE trial, concluding that OS was independent of age, Eastern Cooperative Oncology Group Performance Status (ECOG-PS), KRAS mutational status, and site of metastases at randomization^5^. According to this analysis, factors of good prognosis were low tumor burden and indolent disease when initiating trifluridine/tipiracil. However, several aspects call into question the external validity and applicability of these results in the real world. Pooled estimates from real-life studies reveal several differences in the baseline characteristics of individuals treated in clinical practice vis-à-vis the ideal population of the RECOURSE RCT^6^. Thus, subjects with worse ECOG-PS or more aggressive tumors are routinely treated in the real world. For example, in a series from the Netherlands, patients treated with trifluridine/tipiracil had worse functional status (ECOG PS of 1 or 2) in 57% and 9%, compared to 44% and 0% in the RECOURSE study population, respectively^7^. Similarly, there were more pretreated patients, as well as a greater proportion of KRAS mutated cancers, in comparison with the RECOURSE RCT; both variables were associated with impacting prognosis. Furthermore, this classification is based on bivariate analyses (log rank tests)^5^. Consequently, the additive contribution of multiple variables or information sources on prognosis (e.g., an individual may have begun trifluridin-tipiracil > 18 months, a favorable factor, yet also have a high tumor burden, an unfavorable factor) has not been contemplated. The Colon Life nomogram, a tool to predict prognosis in mCRC^8^ has recently been developed and subsequently validated in the RECOURSE RCT cohort^9^. In addition, the FAS-CORRECT model was devised on the basis of the compassionate use of regorafenib (REBECCA) program in refractory mCRC^10^. These models might help to enhance patient classification.

In this sense, we have sought to externally validate the RECOURSE RCT prognostic factors, as well as the Colon Life nomogram and FAS-CORRECT, in a multicenter, real-world series. Secondarily, we have elaborated the TAS-RECOSMO (TAS-102- trifluridine-tipiracil- in REfractory COlorectal cancer Spanish MOdel) model that makes individualized prediction possible in this population.

## Method

### Patients and study design

The study population proceed from a database to which 12 Spanish hospitals have contributed. The design was a retrospective. Eligibility criteria included age ≥ 18 years, presence of histologically confirmed mCRC, administration of at least one cycle of trifluridine/tipiracil in third or successive lines, and treatment initiation between June 2016 and June 2019. Centers were asked to collect all consecutive cases meeting eligibility criteria. The study was performed in accordance with Good Clinical Practice guidelines and the Declaration of Helsinki. This observational, non-interventional trial was approved by the Research Ethics Committee of all centers that includes Ethics Committee of Galicia, Hospital General Universitario José María Morales Meseguer and Hospital Central de Asturias. All participants still alive at the time of data collection provided written, signed, informed consent. Informed consent and approval by the competent national authorities includes permission for publication and dissemination of the data. The protocol is shown in Supplementary File 1.

### Selection of variables

The primary endpoint principal was OS defined as the time between commencement of trifluridine/tipiracil until death or loss to follow-up. Progression-free survival (PFS) was defined as the interval between beginning trifluridine-tipiracil until progression or demise, right-censoring event-free subjects at the time of last follow-up. Factors for the predictive model were selected after comprehensively reviewing previously published literature^5,11^. The covariates chosen were neutrophil–lymphocyte ratio (NLR, continuous variable), CEA (continuous, non-linear variable), ECOG-PS, number of metastatic sites (organs involved, dichotomized as in Tabernero et al., < 3 vs ≥ 3)^5^, time since diagnosis of metastasis until starting TAS-102 (evaluated continuously and dichotomized as in Tabernero et al., < 18 vs ≥ 18 months), and alkaline phosphatase (continuous, non-linear variable). Tabernero et al. established two groups: cancers with good prognostic characteristics (GPC) defined as neoplasms having a low tumor burden (< 3 metastatic sites) and indolent disease (≥ 18 months from diagnosis of metastatic disease to trifluridine/tipiracil) (RECOURSE groups); the rest were deemed to have poor prognostic characteristics (PPC)^5^. The Colon Life nomogram comprises four variables (ECOG-PS, resection primary tumor, LDH value, and peritoneal involvement); the model was assessed as per the original description^8,9^. The FAS-CORRECT model consists of four variables: ECOG PS (0, 1, ≥ 2), time since initial diagnosis (≥ 18, < 18 months), number of metastatic sites (< 3, 3 +), and liver metastases^10^.

### Statistics

A log-normal accelerated failure time (AFT) model was used, given that several variables exhibited a dynamic effect. This model assumes that the effect of the covariates is to accelerate or decelerate the course of illness, making it suitable when the assumption of proportional hazards is not met^12^. Survival times in AFT models are multiplied by a constant effect under this formulation, such that the exponential coefficients of the model are denominated time ratios (TR). A TR > 1 implies a longer time to event, whereas a TR < 1 means that the events occurred sooner. Thus, a TR equal to log(0.5) represents that the median time to event is halved in its presence. Since this is a non‑interventional, fixed sample size study, inferences should be interpreted according to the magnitude of the CI with a descriptive purpose. The strategy to specify the model was to adopt one degree of freedom for every 15 events available until the highest number of variables ran out, basing decisions on the correlation of the variables with OS (with Somers’ Dxy rank correlations) and comparing nested models by applying the Akaike information criterion (AIC)^13^. Non-linear effects were visually inspected and, when necessary, continuous variables were modelled using restricted cubic splines. Discrimination was evaluated by means of bootstrapped bias-corrected Harrel’s c-index, while 6- and 12-month calibration was evaluated visually. Analyses were performed in R v4.0.4 with the rms, Hmisc, and visreg software packages^14–16^.

## Results

### Patients

Two hundred and forty-four (244) patients were recruited. Baseline characteristics are shown in Table 1. Subjects received trifluridine/tipiracil following a median of 31.0 months (95% CI, 28.4–33.7) from the time of diagnosis of metastasis, and after progression to two or three previous lines (43.0% and 52.8%, respectively). Thirty-two percent (32%) had an ECOG-PS 2–3 and approximately one third had more than two metastatic sites. Trifluridine/tipiracil administration was initiated at full dose (70 mg/m^2^/12 h in 72% (n = 175), a one-step lower dose (55–70 mg/m^2^/12 h) in 24% (n = 59), and at lower dosages (40–55 mg/ m^2^/12 h) in 4% (n = 10). Subjects received a median of 3 cycles (range, 1–16). There was at least one delay in 33% (n = 80); median of delayed cycles was 1 (range, 1–6). In 32% (n = 78), dosage was decreased by at least one step during treatment.

**Table 1:** Baseline characteristics.

	N (%)
Age, mean (range)	66 (18–88)
Sex, female	87 (35.7)
**ECOG PS**	
0	31 (12–7%)
1	179 (73.4%)
2	32 (31.1%)
3	2 (0.8%)
**Time since diagnosis of metastasis until starting trifluridine/tipiracil**	
< 18 months	55 (22.5)
≥ 18 months	189 (77.5)
Surgery of the primary tumor	185 (75.8)
**Tumor location**	
Right	47 (19.3)
Left	173 (70.9)
Rectum	24 (9.8)
KRAS/NRAS mutated	156 (63.9)
BRAF tested	101 (41.3)
Positive	3 (3%)
Number of tumor sites > 2	84 (34.4)
**Location of metastases**	
Lung	168 (68.8)
Lung only	31 (12.7)
Peritoneal	72 (29.5)
Liver	180 (73.8)
Liver only	32 (13.1)
Bone	19 (7.8)
Neutrophil-to-lymphocyte ratio, mean (sd)	3.8 (3.5)
CEA (ng/mL), mean (sd)	308 (818)
Missing	1 (0.4)
Ca 19.9 (U/mL)	1133 (5266)
Missing	135 (55.3)
**Line of trifluridine/tipiracil therapy**	
1–2	10 (4.1)
3	105 (43.0)
> 3	129 (52.8)

ECOG-PS, Eastern Cooperative Oncology Group Performance Status; sd, standard deviation; NLR, Neutrophil–lymphocyte ratio.

### Efficacy and toxicity outcomes

**Figure 1:**
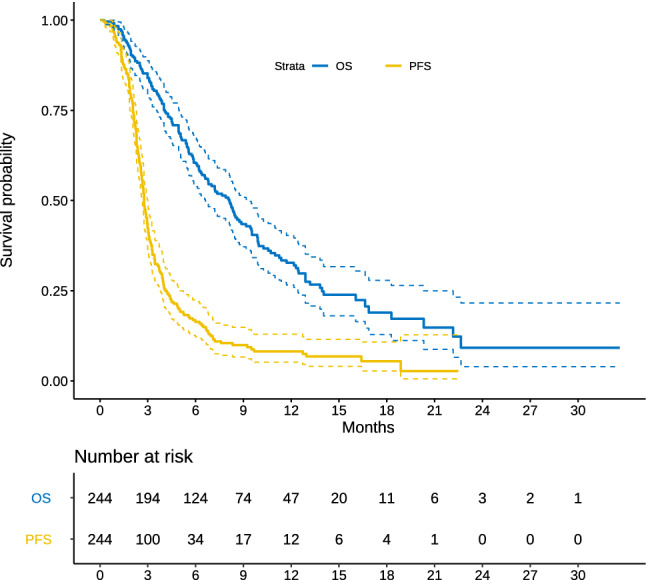
Kapplan Meier OS and PFS curves. Abbreviations: OS, overall survival; PFS, progression-free survival.

**Figure 2:**
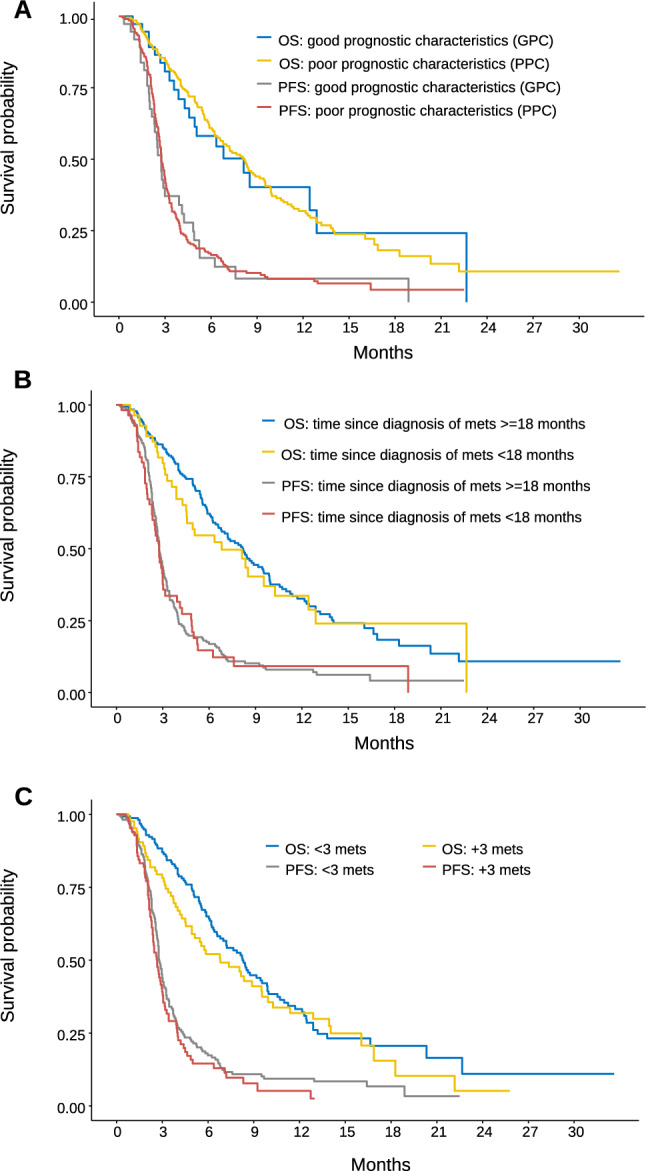
Kapplan Meier OS and PFS curves according to groups of the RECOURSE clinical trial. (**A**) Kapplan Meier OS and PFS curves according to group of good vs poor prognostic characteristics; (**B**) Kapplan Meier OS and PFS curves of indolent (time since diagnosis of metastasis ≥ 18 months) vs aggressive disease (time since diagnosis of metastasis < 18 months); (**C**) Kapplan Meier OS and PFS curves of the groups of low (< 3 metastatic sites) vs high (≥ 3) tumor burden. Abbreviations: OS, overall survival; PFS, progression-free survival; mets, metastases.

Follow-up in living patients was 13.3 months (95% CI, 12.4–14.9). The best response as per RECIST v1.1 was tumor progression in 81% (n = 198), stable disease 13% (n = 32), partial response 2% (n = 4), complete response 0, and not evaluated in 4% (n = 10). At the time of analysis, 218 progression events had been recorded with median PFS of 2.7 months (95% CI, 2.6–2.9) and 161 death events, with median OS of 8.1 months (95% CI, 6.7–9.5) (Fig. 1). PFS/OS Kaplan–Meier curves stratified according to the RECOURSE groups are illustrated in Fig. 2A. Median OS is comparable across both strata (8.15 vs 8.12 months, for poor and good prognosis, respectively; log-rank p = 0.9). Crucially, 85% of the cases were categorized as poor prognosis, whereas 15% had an estimated good prognosis. Figures 2B,C display the curves stratified on the basis of time to beginning trifluridine/tipiracil since diagnosis of metastasis and tumor burden. The relation between treatment effect for OS and time to beginning trifluridine/tipiracil was complex, possibly non-linear, such that, while the initial delay may have been beneficial, it was offset by the increase in the hazard rate at later timepoints.

**Figure 3:**
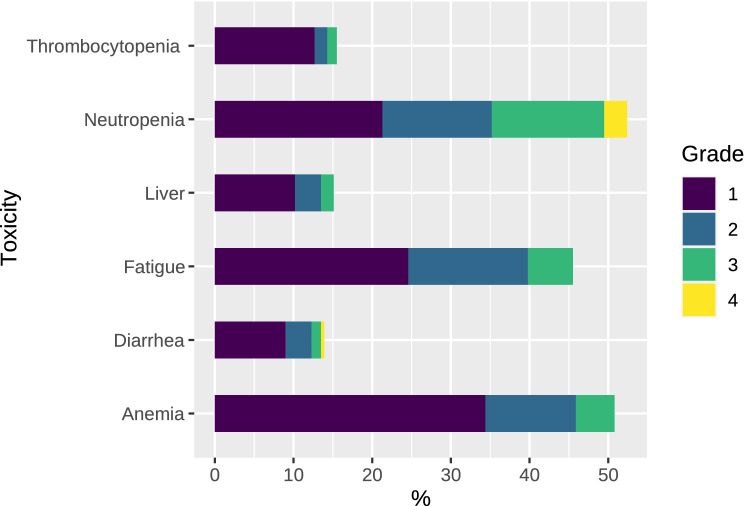
Most common toxicity.

The stacked bars in Fig. 3 summarize the toxicity of trifluridine/tipiracil. Most toxicities were mild (grade 1–2). The most common grade 3–4 toxicity wereeutropenia (17%), asthenia (6%), anemia grade (5%), liver toxicity (2%), and thrombocytopenia (1%).

### AFT model

**Figure 4:**
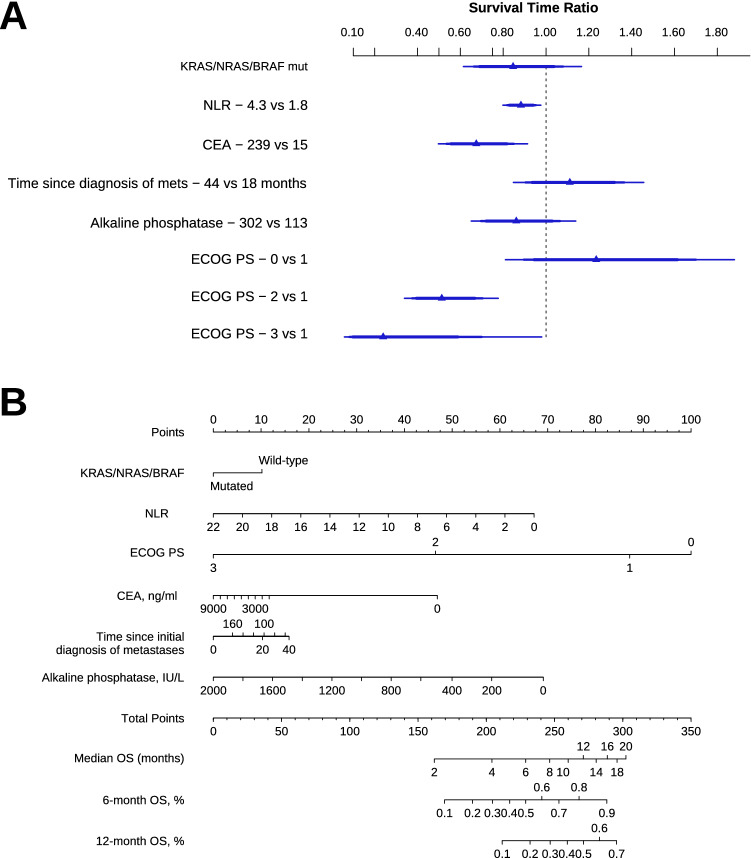
AFT lognormal model for overall survival (**A**), and nomogram (**B**). Abbreviations: AFT, accelerated failure time; NLR, Neutrophil-to-lymphocyte ratio; ECOG-PS, Eastern Cooperative Oncology Group Performance Status; OS, overall survival.

**Figure 5:**
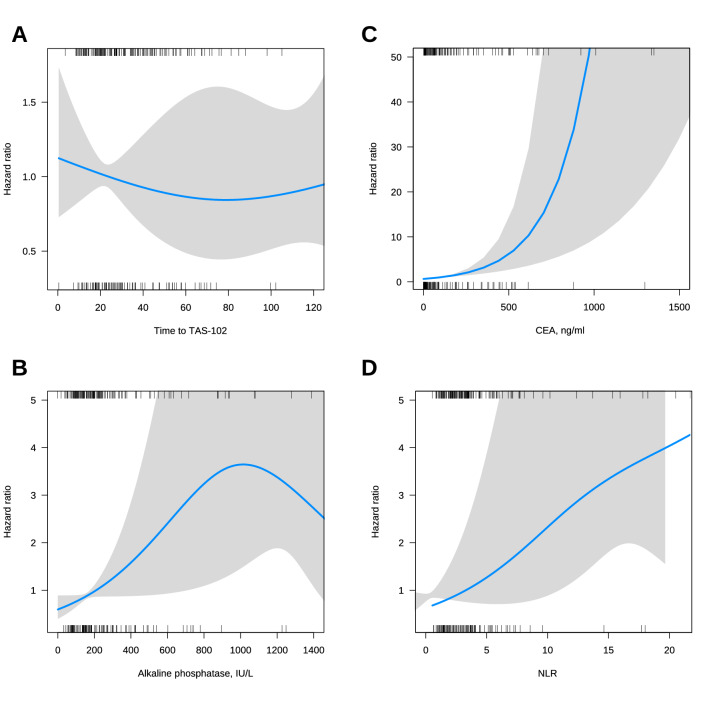
Partial effects of selected variables. Hazard ratio curves allowing non-linear relationships between continuous predictors and overall survival. (**A**) Hazard ratio with non-liner effects for time to TAS-102. (**B**) Hazard ratio with non-liner effects for alkaline phosphatase, IU/L. (**C**) Hazard ratio with non-liner effects for CEA, ng/ml, (**D**) Hazard ratio with non-liner effects for NLR. Abbreviations: NLR, Neutrophil-to-lymphocyte ratio; CEA, Carcinoembryonic Antigen.

We then fitted an AFT lognormal model for OS. TAS-RECOSMO included 6 variables: ECOG-PS, KRAS/NRAS/BRAF mutation status, time since diagnosis of metastasis to initiation of trifluridine/tipiracil, NLR (Fig. 5D), CEA, and alkaline phosphatase (Figs. 4A, B). The prognostic effect of laboratory values, alkaline phosphatase and CEA, was clearly non-linear (Fig. 5B, C), and they turned out to be the variables that most closely correlated with OS (Somers’ Dxy rank correlations in the Data Supplement) (Annex Fig. 1). The indolent vs aggressive course variable quantified by time to trifluridine-tipiracil displayed a slight biphasic pattern so that the initial protective effect was diluted and counteracted at late timepoints (Fig. 5A). Tumor burden, defined as number of metastatic sites, revealed a weak correlation with OS; its inclusion elevated the model’s AIC, and was therefore excluded from the final model. The contrasts for this model are shown in Table 2. TAS-RECOSMO has acceptable discriminatory capacity with bootstrapped bias-corrected c-index of 0.682 (95% CI, 0.636–0.722). The 6- and 12-month calibration plots are illustrated in Annex Fig. 2. The model is well calibrated, except for the range of lowest expected survivals, where OS was slightly overestimated.

**Table 2:** Contrasts of the AFT model lognormal for overall survival.

Factor	Contrast	TR CI 95%
NLR	Continuous	0.88 (0.81–0.95)
CEA, ng/ml	240 vs 15.7	0.67 (0.53–0.85)
Time to trifluridine/tipiracil	44.0 vs 18.5	1.11 (0.90–1.36)
Alkaline phosphatase, IU/L	302 vs 113	0.86 (0.69–1.06)
ECOG PS	1 vs 0	1.23 (0.86–1.76)
	2 vs 1	0.52 (0.38–0.72)
	3 vs 1	0.24 (0.08–0.73)
KRAS/NRAS/BRAF mutation	Yes vs No	0.84 (0.62–1.08)

AFT, accelerated failure time; NLR, Neutrophil–lymphocyte ratio; ECOG-PS, Eastern Cooperative Oncology Group Performance Status; sd, standard deviation; TR, time ratio; CI, confidence interval.

### Comparison with the colon life model, FAS-CORRECT, and RECOURSE

The Colon Life Model, formulated as a proportional hazards model, demonstrated poor calibration, associated with PH assumption violation. Once reformulated as an AFT model, the Colon Life Model yielded a c-index of 0.690 in this series and displayed excellent calibration (Annex Fig. 3), while the FAS-CORRECT model, reformulated as an AFT model, revealed a moderate discriminatory capacity with a c-index of 0.630. By comparison, the AFT model constructed on the RECOURSE groups performed poorly, with a c-index of 0.507, consistent with the absence of discrimination in Kaplan–Meier estimations (Fig. 2).

## Discussion

External validity is the dimension that is overlooked in ranking evidence, since some RCTs may not be representative of the target population or exclude types of patients who do receive the therapy in the real world^17,18^. This pertains to average effects and safety concerns, but is key, inasmuch as it also impacts the capacity to generalize the analyses of prognostic factors to specific populations. In this work, we have evaluated the prognostic factors of patients with mCRC treated with trifluridine/tipiracil.

Our data from clinical practice uphold the external validity and applicability of the outcomes of the RECOURSE RCT, albeit with several nuances. First of all, the survival endpoints are comparable, with a median OS of 7.1 and 8.1 months, and median PFS of 2.0 and 2.7, in the RECOURSE trial and in this study, respectively^2^. Likewise, these results are consistent with other reports of real-world observational studies^19–21^. The response rate is similar, despite differences in the time pattern in chemotherapy administration –every 8 weeks (RECOURSE RCT) and a median of 11 weeks in this series. In the RECOURSE study, 2% partial response and 16% stabilization rates were observed, while in our study, 81% progressed. The administration of trifluridine/tipiracil was likewise feasible; in the RECOURSE RCT it was administered over 12.7 ± 12.0 weeks (median, 6.7; range, 0.1–78.0), whereas here, a median of 3 cycles (range, 1–16) was administered. Our study saw less toxicity compared to the RECOURSE RCT, with less grade 3–4 neutropenia (17% vs 28%), grade 3–4 anemia (5% vs 18%), and less grade 3–4 thrombopenia (1% vs 5%). This appears to be contingent on the use of lower doses to treat unfit individuals and is on a par with other real-world results^19^.

However, the differences are remarkable as they refer to the analysis of prognostic factors. In the RECOURSE RCT, the protracted time to initiation of trifluridine/tipiracil evaluated dichotomously constituted a protective factor, as it was associated with indolent tumors^5^. This was also seen in the Regotas study, where treatment administered < 18 months since diagnosis of metastasis correlated with worse outcomes^22^, albeit not in other studies^23^. Nonetheless, the dichotomization of continuous variables, in this case < 18 vs ≥ 18 months, entails bias and loss of information^24^. In fact, in our study, the timing of administration was similar to that of the RECOURSE RCT, but the results point toward a slight non-linear, continuous effect, with initial protection that is quickly offset by the greater risk with therapies administered after extended periods of time, in advanced phases and cancers. Likewise, tumor burden is a prognostic factor in the RECOURSE RCT and in other series^25^, although it does not appear to be relevant in our study, possibly because trifluridine/tipiracil was administered to a series in which there were many subjects with a high tumor burden, where this variable failed to discriminate between some subjects and others. All this explains why the GPC and PPC groups in the RECOURSE RCT that combine these two factors do not have discriminatory capacity in our clinical practice series.

In contrast, TAS-RECOSMO consists of 6 variables with known prognostic effect. ECOG-PS was not a prognostic factor in the RECOURSE RCT, although it is in the Colon Life nomogram, as it is in this and other series^9,10,22^. Similarly, ECOG-PS, RAS/BRAF mutations, and CEA emerge in a prognostic model for mCRC^11^. NLR is a marker of a pro-inflammatory state and known to be prognostic in colon cancer and other tumors^26,27^. Other predictive factors in the literature are bone metastases, albumin or AST, or platelets^22,25^. Our study yields no evidence that the number of previous lines contributes to prognosis, unlike other series^20,28^, perhaps due to the predominance of 3th or 4th line treatments in our sample.

Overall, the performance of the RECOURSE RCT groups was low in our dataset, possibly because their discriminatory capacity is tied to the population in which the model was elaborated. Their baseline characteristics are more homogenous than those of real-world series, as they are selected according to the strict inclusion criteria of RCTs with ECOG 0–1, laboratory variables within a pre-established range, and good liver and kidney function. This suggests that part of the information about prognosis in clinical practice is found in subjects who were not recruited in the RECOURSE RCT^2^. The percentage of patients with ECOG-PS 0 and 2–3 is 57% and 0 in the RECOURSE RCT, compared to 13% and 32% in our series. Likewise, 48% of the RECOURSE RCT population were included in the GPC group, *versus* 15% in the TAS-RECOSMO. Likewise, KRAS mutations occurred in 51% and 63%, respectively.

In contrast, the Colon Life nomogram is a valid model in our series, with the distinction that the dynamic effect of its variables required that it be reformulated as an AFT model to conserve suitable calibration over time. The FAS-CORRECT is also a valid model, regardless of it having originally been fitted for a cohort of individuals treated with regorafenib^10^. This is not surprising in that the covariates that comprise it are not specific to antiangiogenic therapies.

Readers must be aware of the limitations of our study, the most salient being that TAS-RECOSMO must be externally validated by other groups before it can be recommended for widespread use, although the internal validation suggests that it performs comparably to the Colon Life nomogram and FAS-CORRECT. As far as causal inference is concerned, the small sample size precludes the analysis of multiple variables (restricted by the effective sample size in 15 events per degree of freedom^13^) and increases the uncertainty of some estimations. For instance, KRAS/NRAS/BRAF mutations appear to lower OS by 16%, although the CI is broad, which is also compatible with a smaller magnitude of effect. Finally, the discrepancies in toxicity may be accounted for by the retrospective nature of data collection in this registry, unlike the RECOURSE RCT.

In conclusion, we have developed and internally validated a model, TAS-RECOSMO, that predicts prognosis on the basis of six clinical-pathological and laboratory variables (general status; neutrophil–lymphocyte ratio; KRAS, NRAS and BRAF mutational status; CEA; alkaline phosphatase, and time between diagnosis of metastases until start of trifluridine/tipiracil). Additionally, we have externally validated another two models, the Colon life nomogram and FAS-CORRECT, that could predict OS in individuals with mCCR initiating trifluridine/tipiracil in the real world. The RECOURCE RCT prognostic groups failed to exhibit validity in this series, given that the two variables (tumor burden and indolent/aggressive disease) do not appear to capture the situation of patients treated with trifluridine/tipiracil in the real world. Our data speak to the importance of externally validating the prognostic outcomes obtained in RCTs in patient populations who receive the treatments and scantly resemble those of the trial.

## Supplementary Information

Supplementary figures.

Supplementary Information 2.

## Data Availability

The details of analyses used in the current study are available from the first author or corresponding author upon request.

## Code availability

The R Code is available upon request to the authors.
