# Supplementary material for: Prediction of survival in patients with advanced, refractory colorectal cancer in treatment with trifluridine/tipiracil: real-world vs clinical trial data
Source: Sci Rep. 2021 Jul 12;11:14321. doi: 10.1038/s41598-021-93732-5 (PMC8275736; doi:10.1038/s41598-021-93732-5)

Annex Figure 1. Somers' Dxy rank correlation between predictors and overall survival

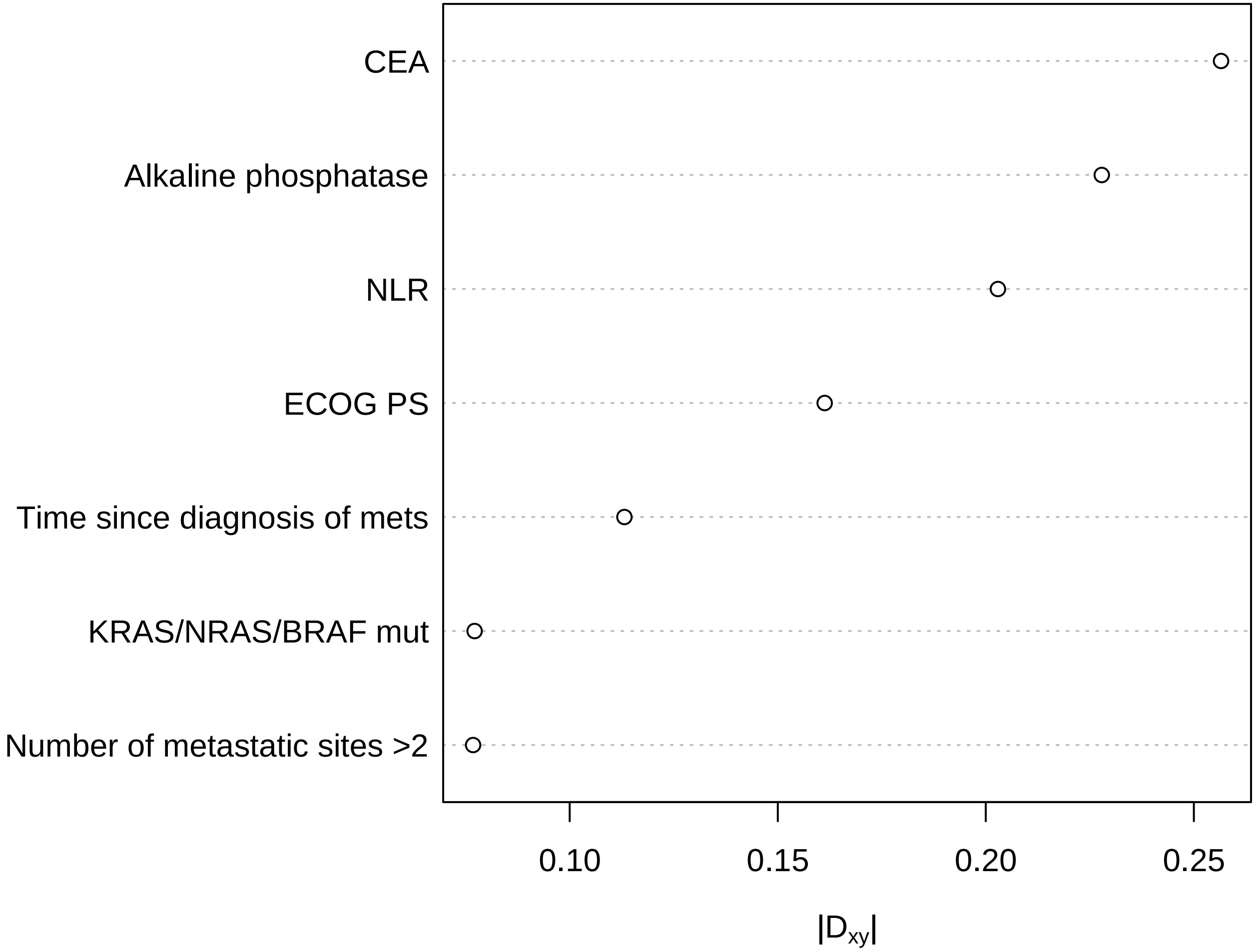

Abbreviations: NLR, Neutrophil-lymphocyte ratio; ECOG-PS, Eastern Cooperative Oncology Group Performance Status; CEA, carcinoembryonic antigen; mets, metastases, mut, mutations

Annex Figure 2. Hazard ratio plots with non-linear effects

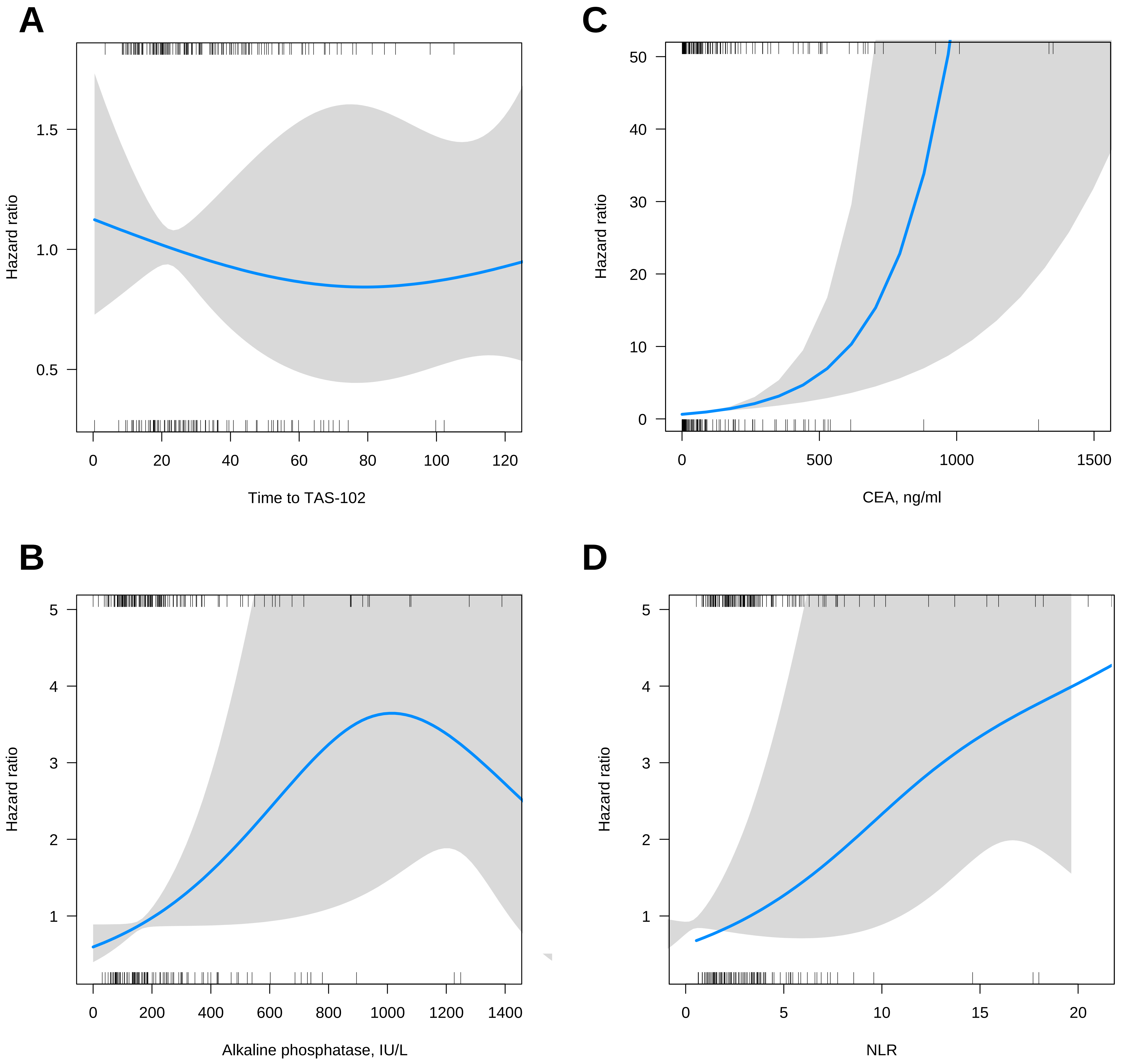

Annex Figure 3. Calibration curves for 6- and 12-month survival

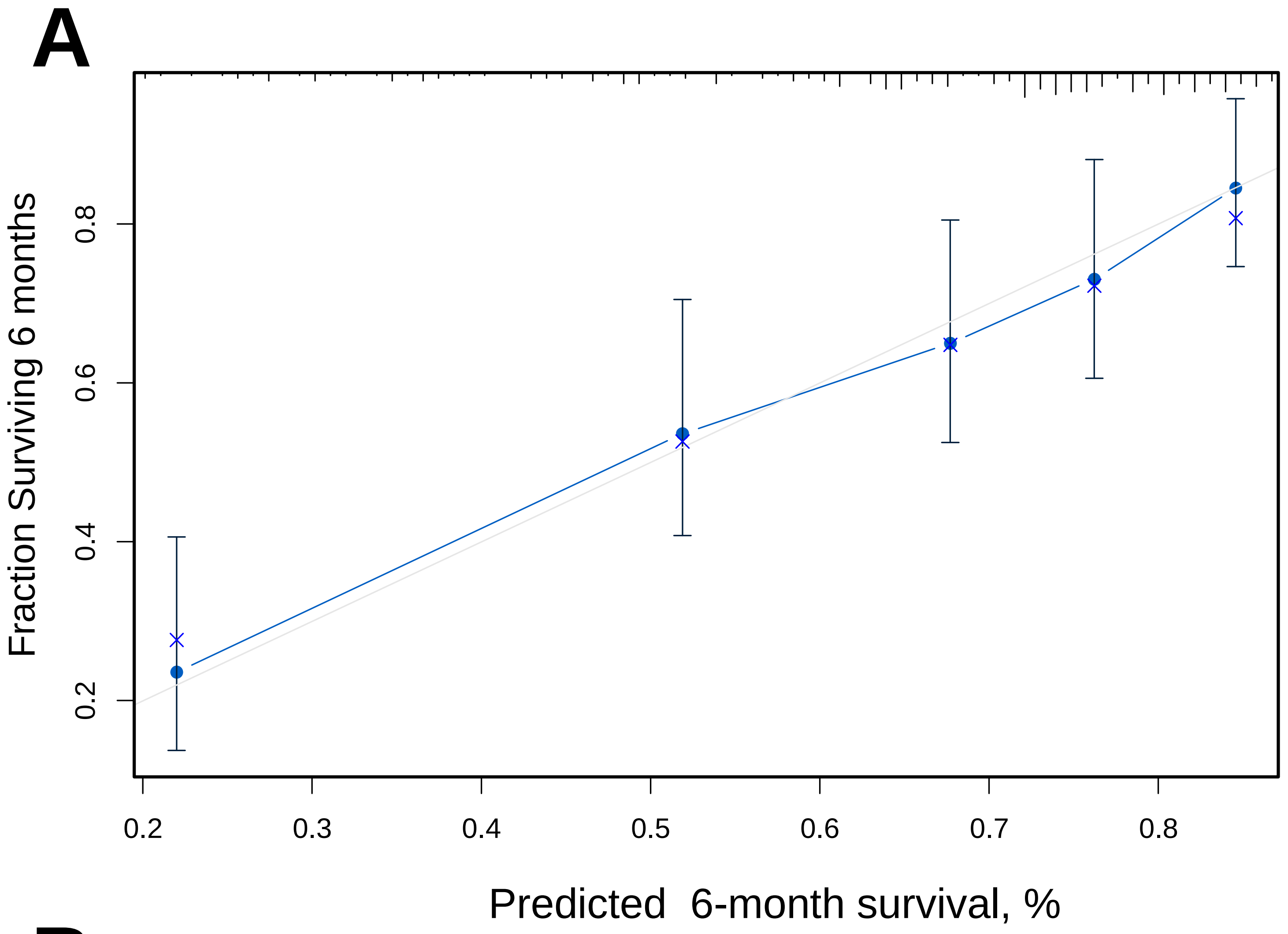

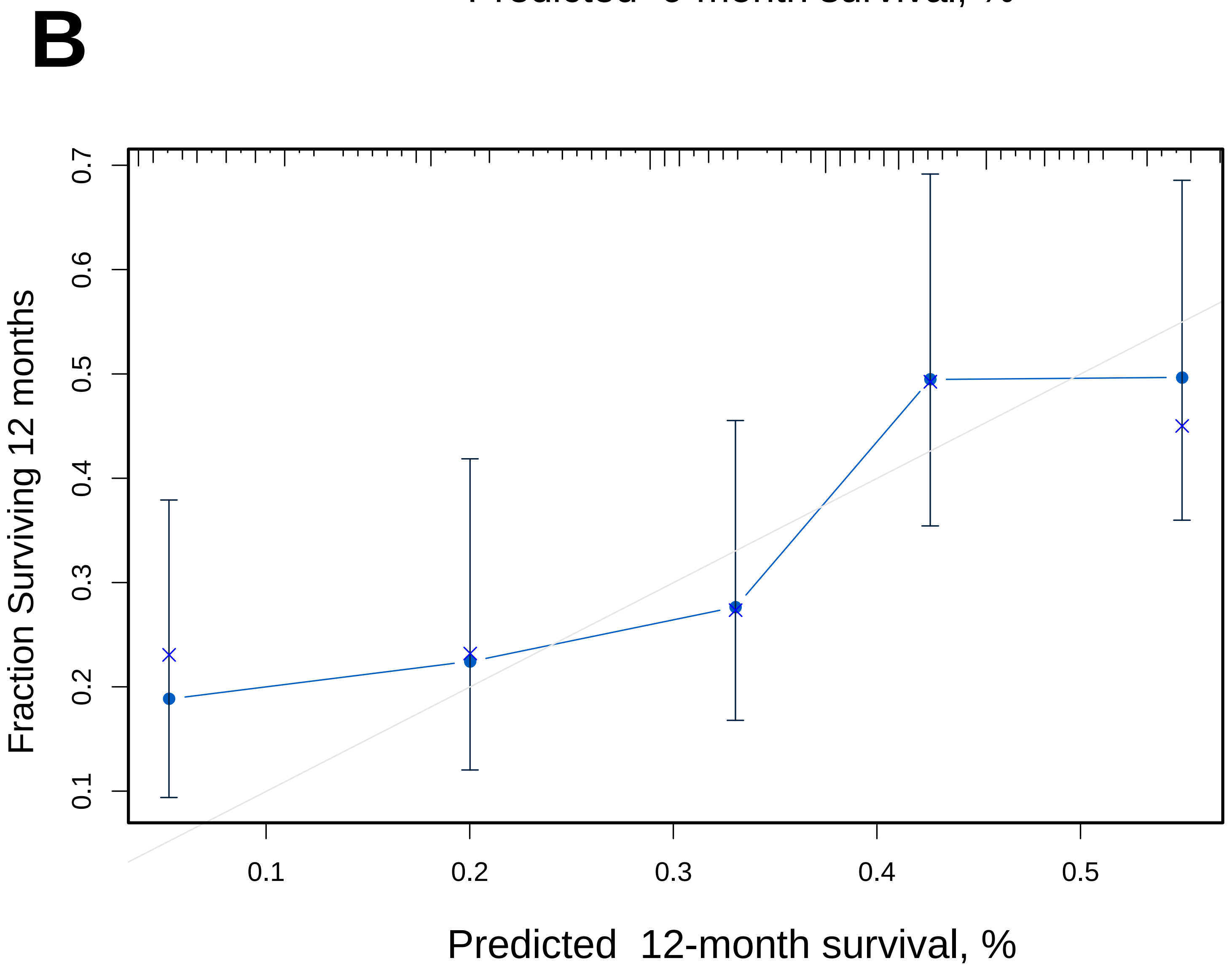

Annex Figure 4. Calibration plots for the Colon Life Model (AFT reformulation)

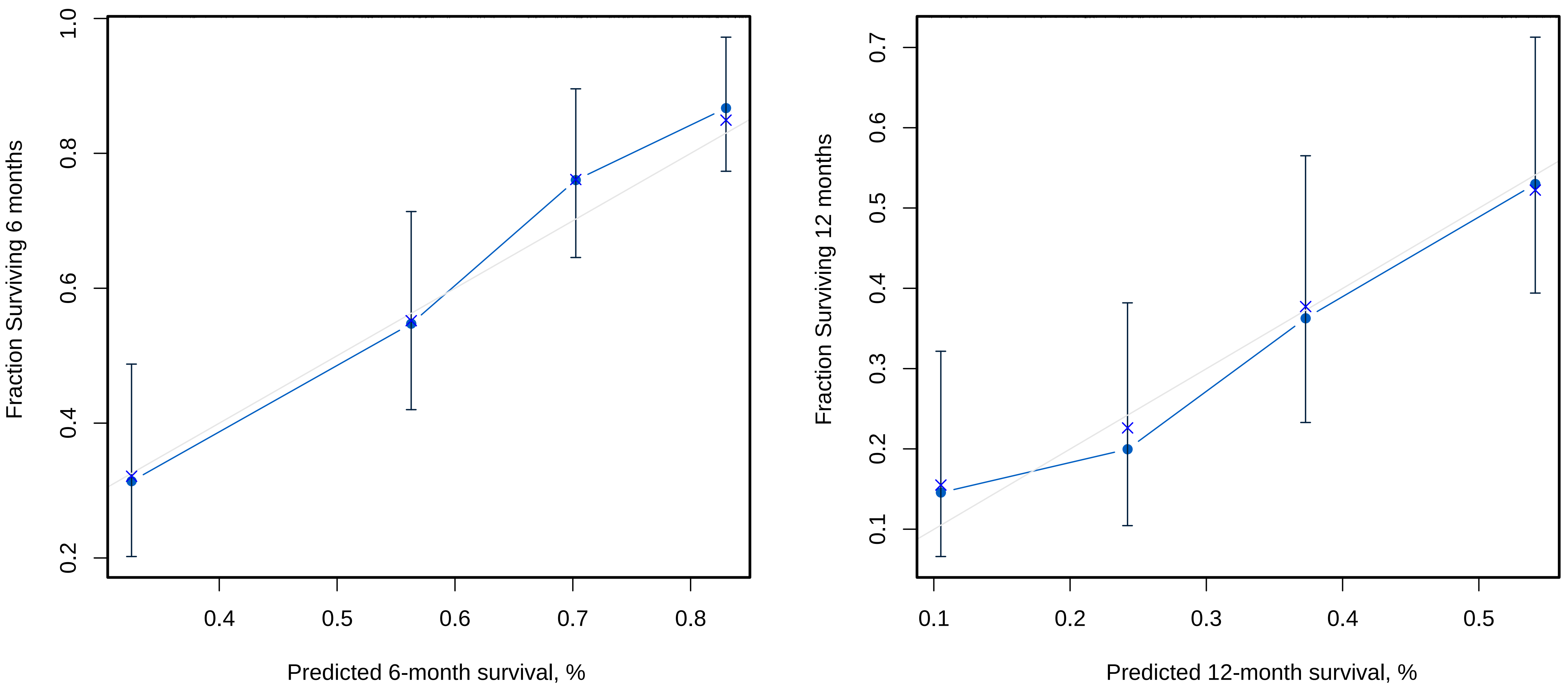

Supplement: Supplementary file 1 — Supplementary figures. [file 41598_2021_93732_MOESM1_ESM.pdf]
