# Supplementary material for: Prediction of survival in patients with advanced, refractory colorectal cancer in treatment with trifluridine/tipiracil: real-world vs clinical trial data
Source: Sci Rep. 2021 Jul 12;11:14321. doi: 10.1038/s41598-021-93732-5 (PMC8275736; doi:10.1038/s41598-021-93732-5)
Supplement: Supplementary file 2 — Supplementary Information 2. [file 41598_2021_93732_MOESM2_ESM.pdf]

Observational, retrospective study to assess the efficacy and safety of Trifluridine / Tipiracil for the treatment of patients with metastatic colorectal cancer in routine clinical practice .

Developer code: GIT-LONS-2018-02

Developer: GITuD

Study phase: Post-authorization, observational, retrospective study

Protocol Number / Version / Date : Version 1 Date February 8 , 2018 .

CONFIDENTIAL

The information and data included in this protocol is the property of the promoter. No person is authorized to make such information public without the promoter's written permission. These restrictions will apply equally to all information that is provided to you in the future and is considered privileged or confidential. This material may be disclosed and used by your team and associates, insofar as it may be necessary for the development of the clinical study.

PROMOTER SIGNATURE SHEET

I have read this protocol and approve the study design

INDEX

SUMMARY 6

1. GENERAL INFORMATION 10

STUDY IDENTIFICATION 1 0

DETAILS OF THE PROMOTER 10

MONITORING 10

RESEARCHERS AND CENTERS WHERE THE STUDY IS PLANNED TO BE CONDUCTED 10

2. INTRODUCTION AND JUSTIFICATION OF THE STUDY 10

INTRODUCTION 10

Colorectal Cancer 10

Treatment of metastatic colorectal cancer (mCRC) 10

Trifluridine-Tipiracil in the treatment of metastatic colorectal cancer 11

JUSTIFICATION OF STUDY 12

3. OBJECTIVES OF THE STUDY 13

MAIN GOAL 13

SECONDARY OBJECTIVES 13

4. DESIGN AND STUDY POPULATION 14

TYPE OF STUDY 14

STUDY POPULATION 14

Inclusion criteria 14

Exclusion criteria 14

INFORMATION SOURCE 14

EXPECTED SIZE OF THE SAMPLE AND BASIS FOR THEIR DETERMINATION 15

ASSESSMENT VARIABLES OF THE STUDY 15

Main Variable 15

Secondary variables 15

DESCRIPTION OF TREATMENT AND DEFINITION OF EXPOSURE 15

5. DATA MANAGEMENT AND STATISTICAL ANALYSIS 15

METHODS FOR DATA COLLECTION 1 5

STATISTICAL DATA ANALYSIS 16

DATA MANAGEMENT AND QUALITY CONTROL 17

6. ETHICAL ASPECTS 17

GENERAL ETHICAL ASPECTS 17

EVALUATION BY THE CLINICAL RESEARCH ETHICS COMMITTEE 18

Modifications to protocol 18

BENEFIT-RISK ASSESSMENT FOR PARTICIPATING SUBJECTS 18

INFORMATION SHEET AND INFORMED CONSENT 18

DATA CONFIDENTIALITY 19

INTERFERENCE WITH PHYSICIAN'S PRESCRIBING HABITS 20

7. MANAGEMENT AND NOTIFICATION OF ADVERSE EVENTS / ADVERSE REACTIONS 20

SAFETY INSTRUCTIONS 20

Obligations of the Physician regarding the presentation of safety reports 21

SAFETY REMARKS 22

8. PLANS FOR THE DISSEMINATION OF THE RESULTS 22

FOLLOW-UP AND FINAL REPORTS 22

9. RESOURCES FOR THE REALIZATION OF THE STUDY AND ASSIGNMENT OF TASKS. FINANCING 23

10. REFERENCES 24

11. APPENDICES 26

APPENDIX 1. DATA COLLECTION NOTEBOOK 26

ABBREVIATIONS

5-FU	5-fluorouracil
AA	Adverse event
AAG	Serious adverse event
AEMPS	*Spanish Agency for Medicines and Health Products*
PCB	Good clinical practice
CCAA	Autonomous communities
CCR	Colorectal cancer
CCRm	Metastatic colorectal cancer
CEIC	Ethical Committee for Clinical Research
CRD	Data collection notebook
CRDe	Electronic data collection notebook
CRO	Contract Research Company
ECOG	Eastern Cooperative Oncology Group
EGF	Endothelial growth factor (*Epidermal Growth Factor*)
FOLFIRI	irinotecan / 5-fluorouracil / folinic acid
ICH	International Harmonization Conference (*International Conference on Harmonization*)
MedDRA	Medical Dictionary for Regulatory Activities
NCI-CTCAE	Common terminology criteria for the classification of Adverse events of the National Cancer Institute (*National Cancer Institute Common Terminology Criteria for Adverse Events*)
PIGF	Placental growth factor
RECIST	Response Evaluation Criteria In Solid Tumors
RO	Objective responses
SG	Overall survival
SLP	Progression Free Survival
SOC	Organ systems (*System organ class*)
TAC	Marketing authorization holder
VEGF	*Vascular* endothelial growth factor (*Vascular Endothelial Growth Factor)*

RESUME

1. Type of request

Post-authorization, observational, retrospective study with an active principle of a pharmaceutical specialty under authorized conditions of use.

2. Study title

Observational, retrospective study to assess the efficacy and safety of Trifluridine / Tipiracil for the treatment of patients with metastatic colorectal cancer in routine clinical practice .

3. Protocol code

Developer code: GIT-ADE-2018

4. Release date : February 8 , 2018

5. Version number : 1.0

6. Promoter : Galician Group for Research in Digestive Tumors (GITuD)

7. Monitoring : No p r ocede

8. Project phase

Post-authorization, observational, retrospective and Galician study (9 centers) .

9. Researchers and centers where the study is planned

The study will be carried out in the oncology services of the following Hospitals in Spain:

- University Hospital Complex of Ourense (Oure nse)
- Santiago de Compostela Clinical Hospital (Santiago)
- A Coruña Cancer Center (A Coruña)
- Lucus Augusti University Hospital (Lugo)
- Marcide Architect Hospital (Ferrol)
- Juan Canalejo Hospital (A Coruña)
- University Hospital of Pontevedra (Pontevedra)
- Povisa Hospital (Vigo)
- Álvaro Cunqueiro Hospital (Vigo)
- Central Hospital of Asturias (Oviedo)
- Morales Messeguer Hospital (Murcia)

10. CEIC

The clinical trial protocol will be evaluated by an edited Clinical Research Ethics Committee (CEIC) .

11. Objectives of the Study

Main goal

- To assess the efficacy of Trifluridine / Tipiracil in real conditions of use in patients with mCRC (effectiveness in terms of PFS).

Secondary objectives

- To assess the efficacy of Trifluridine / Tipiracil in real conditions of use in patients with mCRC (effectiveness in terms of response rates, overall survival).
- To evaluate the safety profile of Trifluridine / Tipiracil in real conditions of use in patients with mCRC.
- Evaluate the exposure to the treatment and its duration.
- To evaluate the specific weight of the tumor location (right or left colon), RAS status, primary resection, overall survival and progression-free survival as prognostic factors.
- To assess the correlation of the incidence of neutropenia with efficacy variables such as PFS, OS, and response rate.
- Analyze PFS and OS based on the number of metastatic sites, ECOG , tumor location (right, left) and progression-free interval to the last line of treatment prior to Trifluridine / Tipiracil.
- Evaluate the growth modulation index (GMI), as a variable to determine the effectiveness of a third line.
- Design a lonsurf efficacy predictive nomogram.

12. Main variable

- Effectiveness of Trifluridine / Tipiracil measured in terms of PFS

Secondary variables

- Effectiveness of Trifluridine / Tipiracilo measured in terms of:

• Percentage of objective response rates (OR) according to the RECIST v1.1 criteria.

• Overall survival (OS)

- Collection of AAs evaluated according to the criteria of the Common Terminology Criteria for the classification of adverse events of the National Cancer Institute (NCI-CTCAE) v4.0 and classified according to MedDRA terminology.
- Exposure to Trifluridine / Tipiracil : Number of cycles received, cumulative dose, dose reductions .
- Reasons for termination of treatment.
- Before and after treatment received.

13. Design l clinical study

Study post-authorization observational, multicenter, retrospective analysis of a cohort of patients with MRCC, who have been previously treated or are not considered candidates for treatment with available therapies, including chemotherapy fluoropyrimidine - based, oxaliplatin and irinotecan, agents anti- VEGF and anti-EGFR agents and has yan received Trifluridine / Tipiracilo according to routine clinical practice .

Patients who received Trifluridine / Tipiracil treatment for mCRC within routine clinical practice will be included in the study .

As it is an observational and retrospective study, data will be collected from the clinical history of each patient in each center since the introduction of treatment with Trifluridine / Tipiracil . There will be no prospective follow-up of the patient.

Eligible patients will grant their consent to participate in the study, and to allow their hi storias clinics are reviewed. The ethics committee will be asked to exempt the signing of the informed consent in those patients already deceased at the beginning of the study.

14. Disease under study

Adults with mCRC who have been previously treated or are not considered candidates for treatment with available therapies, including chemotherapy based on fluoropyrimidines, oxaliplatin and irinotecan, anti-VEGF agents and anti-EGFR agents.

15. Description of treatment

Trifluridine-Tipiracil (Lonsurf ®) in routine clinical practice

16. Study population and total number of subjects:

Information from approximately 60 patients with mCRC will be retrospectively reviewed .

17. Eligibility and Withdrawal Criteria

Inclusion criteria:

1. Patients with a diagnosis of mCRC who have been previously treated or are not considered candidates for treatment with available therapies, including chemotherapy based on fluoropyrimidines, oxaliplatin and irinotecan, anti-VEGF agents and anti-EGFR agents and have received treatment from 3rd line with Trifluridine / Tipiracil
2. ECOG 0-2.
3. Age ≥18 years
4. Patients who have given their informed consent, preferably in writing, or orally before independent witnesses of the research team after having been informed and having had enough time to decide about their inclusion in the study.

Exclusion criteria:

Patients must not have received any other investigational drugs or other cancer treatments during treatment with Trifluridine / Tipiracil , including: chemotherapy, immunotherapy, biologics, and hormonal treatments.

Study duration

• Presentation to CEIC

• Data collection period (information from medical records): 3 months.

• Expected closing date of the study and report of results : December 2018.

1. GENERAL INFORMATION

Study identification

Title: Observational, retrospective study to assess the efficacy and safety of Trifluridine / Tipiracil for the treatment of patients with metastatic colorectal cancer in routine clinical practice .

Promoter Information : Not applicable

Researchers and centers where the study is planned

The study will be carried out in the oncology services of the following Galician hospitals :

- University Hospital Complex of Ourense (Ourense)
- Santiago de Compostela Clinical Hospital (Santiago)
- A Coruña Cancer Center (A Coruña)
- Lucus Augusti University Hospital (Lugo)
- Marcide Architect Hospital (Ferrol)
- Juan Canalejo Hospital (A Coruña)
- University Hospital of Pontevedra (Pontevedra)
- Povisa Hospital (Vigo)
- Álvaro Cunqueiro Hospital (Vigo)
- Central Hospital of Asturias (Oviedo)
- Morales Messeguer Hospital (Murcia)

Introduction

Colorectal cancer (CRC) is the third most diagnosed cancer in men (746,000 cases, 10% of the total) and the second in women (614,000 cases, 9.2% of the total) worldwide, with more than 1.4 million new cases diagnosed in 2012. CRC is one of the cancers with the highest mortality and was the cause of more than 600,000 deaths worldwide in 2012 alone (1). In Europe, CRC is the most common cancer in the general population, and is the second leading cause of death due to cancer. In 2012, there were 447,000 new diagnosed cases and 215,000 deaths from CC R (2). In Spain, the CCR occupies the first position and the estimate of the number of new cases for the year 2015 was 41,441 cases (3)

Approximately 20% of patients present metastatic disease at the time of diagnosis and a significant proportion of those who have been previously treated will experience a recurrence of the disease, developing metastases mainly in the liver and lungs (4). Despite the fact that in the last 5 years the introduction of new drugs and the optimization of regimens with 5-Fluorouracil (5-FU) has allowed the overall survival to be almost doubled, in patients with metastatic disease survival at 5 years is still around 12% (5) .

The treatment of metastatic colorectal cancer (mCRC) involves the use of active cytotoxic drugs (irinotecan, oxaliplatin, 5-fluorouracil [5-FU], and capecitabine) both in combination and in monotherapy. The addition of irinotecan and oxaliplatin to regimens based on 5-FU and leucovorin are the reference treatments in the first and second line of mCRC or advanced (4, 6-9).

In the last decade , therapeutic strategies in mCRC have focused on the addition of monoclonal antibodies to conventional cytotoxic therapy, such as antibodies against vascular endothelial growth factor (VEGF) and antibodies against endothelial growth factor receptor (EGFR) . Currently, anti-VEGF therapies available for the treatment of mCRC include the monoclonal antibody bevacizumab, which binds to VEGF-A, and the monoclonal antibodies cetuximab and panitumumab, which indirectly inhibit angiogenesis by targeting the endothelial growth factor receptor. (EGFR). With the addition of these drugs has improved the s rate s response and survival when combined with chemotherapy for first- or second - line treatment of mCRC.

Regorafenib (10) and Trifluridine / Tipiracil (11) are two new drugs that have an indication in patients who have progressed to chemotherapeutic drugs, irinotecan, oxaliplatin, fluopyrimidines, anti-VEGF agents and anti-EGFR agents .

As treatment options continue to increase for mCRC , the selection of the most appropriate therapeutic regimen and sequence of administration , as well as its integration with other treatment modalities (eg surgery), become increasingly complex. . Since the choice of first-line treatment will compromise later treatment options, it is important to plan in advance a tentative therapeutic strategy, particularly in those patients with unresectable disease (12,13).

Trifluridine / Tipiracil (Lonsurf) is an orally administered drug combination of a thymidine-based nucleic acid analog, trifluridine, and a thymidine phosphorylase inhibitor, Tipiracil hydrochloride. Trifluridine is the active cytotoxic component of Trifluridine / Tipiracil . Its triphosphate form binds to DNA, and this binding is responsible for the antitumor effect (14). Tipiracil hydrochloride is a potent thymidine phosphorylase inhibitor and, when combined with trifluridine, prevents the rapid degradation of trifluridine, allowing the maintenance of adequate plasma levels of the active drug (15).

Preclinical studies have shown that Trifluridine / Tipiracil has antitumor activity in cell lines resistant to fluorouracil (16,17). The results of phase I clinical trials (18-22) have determined that Trifluridine / Tipiracil is effective and safe when administered in cycles of 28 days, where each cycle consists of 5 days of treatment , two days off, 5 days more treatment followed by a 14-day rest period. Based on these studies the recommended dose was 35 mg / m2 twice daily.

Trifluridine / Tipiracil was subsequently evaluated in a phase II, double-blind, randomized, placebo-controlled trial of 169 Japanese patients with metastatic colorectal cancer refractory to fluorouracil, both irinotecan and oxaliplatin (23). Median overall survival was 9.0 months in the Trifluridine / Tipiracil group and 6.6 months in the placebo group (hazard ratio of death, 0.56, P = 0.001).

Finally conducted a Phase III (11) to evaluate the efficacy and safety of Trifluridine / Tipiracilo in an overall population of 800 patients with mCRC who had been refractory s to antitumor therapy or had n I had adverse events clinically significant that prevented the re-administration of these treatments. In this trial the median overall survival improved from 5.3 months with placebo to 7.1 months with Trifluridine / Tipiracil and the risk ratio of death in the Trifluridine / Tipiracil group *vs* the placebo group was 0.68 (CI 95%, 0.58 to 0.81, P <0.001). Trifluridine / Tipiracil , compared to placebo, was associated with a significant improvement in overall survival. In this study , the incidence of adverse reactions was 85.7% (457/533 cases). The most frequently observed clinically significant adverse events associated with Trifluridine / Tipiracil were neutropenia, which occurred in 38% of those treated, and leukopenia, which occurred in 21%. 4% of patients receiving Trifluridine / Tipiracil had febrile neutropenia and one Trifluridine / Tipiracil- related death was reported .

Study justification

Randomized clinical trials are considered the benchmark for comparing the efficacy and safety of treatments. However, these studies do not always answer the questions posed by the clinician in healthcare practice, since they are implemented with very rigorous selection criteria, which ultimately restricts the generalizability of their conclusions. These concepts underline the need for observational studies that answer unresolved questions and complete the information from clinical trials, helping to improve knowledge about the actual use of drugs after marketing.

Additionally, studies carried out under normal clinical conditions allow us to find out if the usual patterns of use by the clinician correspond to the authorization, which is based in turn on the information obtained from clinical trials. Trifluridine / Tipiracil has been approved for use in patients with metastatic colorectal cancer (mCRC), who have been previously treated or are not considered candidates for treatment with available therapies, including chemotherapy based on fluoropyrimidines, oxaliplatin and irinotecan, anti-VEGF agents. and anti-EGFR agents.

Therefore, the aim of the study is to evaluate the efficacy and safety of Trifluridine / Tipiracil in the real-life setting, with patients in routine clinical practice . Information from these patients treated in real life outside of clinical trials will be invaluable in understanding the efficacy and safety of treatment in a less restrictive screening setting compared to the clinical trial.

2. OBJECTIVES OF THE STUDY

Main goal

- To evaluate the efficacy of Trifluridine / Tipiracil in real conditions of use in patients with mCRC (effectiveness in terms of PFS).

Secondary objectives

- To assess the efficacy of Trifluridine / Tipiracil in real conditions of use in patients with mCRC (effectiveness in terms of response rates, overall survival).
- To evaluate the safety profile of Trifluridine / Tipiracil in real conditions of use in patients with mCRC.
- Evaluate the exposure to the treatment and its duration.
- To evaluate the specific weight of the tumor location (right or left colon), RAS status, primary resection, overall survival and progression-free survival as prognostic factors.
- Analyze PFS and OS based on the number of metastatic sites, ECOG, tumor location (right, left) and progression-free interval in the last line of treatment prior to Trifluridine / Tipiracil.
- Evaluate the growth modulation index (GMI), as a variable to determine the effectiveness of a second line.
- Analyze the relationship of the development of neutropenia with efficacy variables such as PFS, SG and TR.

3. DESIGN AND STUDY POPULATION

Type of study

Observational, multicenter, retrospective analysis study of a cohort of patients with mCRC, who have been previously treated or are not considered candidates for treatment with available therapies, including chemotherapy based on fluoropyrimidines, oxaliplatin and irinotecan, anti-VEGF agents and anti-VEGF agents. -EGFR and who have received treatment with Trifluridine / Tipiracil within the usual clinical practice

As it is an observational and retrospective study, patient data will be collected from each center from their clinical history since the introduction of treatment with Trifluridine / Tipiracil . There will be no prospective follow-up of the patient.

Eligible patients will consent to participate in the study, and to allow their medical records to be reviewed. The ethics committee will be asked to exempt the signing of the informed consent in those patients already deceased at the beginning of the study.

Study population

Inclusion criteria:

1- Patients with a diagnosis of mCRC who have been previously treated or are not considered candidates for treatment with available therapies, including chemotherapy based on fluoropyrimidines, oxaliplatin and irinotecan, anti-VEGF agents and anti-EGFR agents and have received treatment from 3rd line with Trifluridine / Tipiracil

2- ECOG 0-2

3- Age ≥18 years

4- Patients who have given their informed consent, preferably in writing, or orally before independent witnesses of the research team after having been informed and having had enough time to decide about their inclusion in the study.

Exclusion criteria:

Patients must not have received any other investigational drugs or other cancer treatments during treatment with Trifluridine / Tipiracil, including: chemotherapy, immunotherapy, biologics, and hormonal treatments.

Source of information

All the data to be collected are obtained as part of the routine of the usual care control and as such appear in the corresponding medical records. All 60 patients will be included .

Expected sample size and basis for its determination

This is a retrospective data collection to assess the efficacy and safety profile of Trifluridine / Tipiracil in mCRC . S and collect information from all patients cum plan the inclusion criteria, which are estimated a sample size of 60 patients

Study endpoints

Main Variable

- Effectiveness of Trifluridine / Tipiracil measured in terms of PFS

Secondary variables

- Effectiveness of Trifluridine / Tipiracilo measured in terms of:
  - Percentage of objective responses (OR) according to the RECIST v1.1 criteria.
  - Overall survival (OS)
- Collection of AAs evaluated according to the criteria of the Common Terminology Criteria for the Classification of Adverse Events of the National Cancer Institute (NCI-CTCAE) v4.0 and classified according to MedDRA terminology.
- Exposure to Trifluridine / Tipiracil : Number of cycles, cumulative dose and dose reductions.
- Reasons for termination of treatment.
- Previous and subsequent treatments received.

Description of Treatment and definition of Exposure

Trifluridine / Tipiracil (Lonsurf ®)

5. DATA MANAGEMENT AND STATISTICAL ANALYSIS

Methods for obtaining data

All the data collected will correspond to patients who have given their consent, preferably in writing, or orally before independent witnesses of the research team. The data collected will correspond to those usually collected in routine clinical practice, and include demographic data of the patients, routine laboratory values required in the diagnosis and control of this pathology, functional status, clinical status, use of medication, tolerability, adverse events, and compliance with treatment (reported by patient and physician). The data will be collected by each researcher in Excel sheets and jointly analyzed using the SPSS program .

The following data will be collected from the review of the patient's medical history :

Patient characteristics : Demographic data (date of birth, sex, race, height, weight), and relevant medical history , ECOG performance status, associated medical conditions, and treatment history .

Tumor Characteristics : History of mCRC (date of initial diagnosis, histology, tumor classification, date of diagnosis of metastatic disease, tumor location , primary surgery or not, and metastasis location (synchronous / metachronous tumor; use of adjuvant) prior). State of the K-RAS. Percentage of RO mutation, according to RECIST version 1.1 Date progression, death or last contact to assess PFS, time to progression and data SG to the year after starting treatment.

Treatment with Triluridine / Tipiracil: (date of first / last dose, initial dose and possible modifications and interruptions, reason for definitive discontinuation [progression, death, AA, others]. In case of temporary interruption: duration and reason for withdrawal temporary (AA, others). AA of all grades and SAA that occur with treatment with Trifluridine / Tipiracil (identification, start and end date, grade or intensity). Successive treatments for metastatic disease (if available) if available. of progression Previous treatments for metastatic disease.

Statistical analysis of the data

The data analysis will be carried out using SPSS, version 9 or higher.

A Statistical Analysis Plan will be carried out that will contain in detail the tests to be carried out as well as the necessary assumptions.

The analysis given the nature of the study will be descriptive:

- The qualitative variables will be described by the absolute and relative percentage frequencies.
- In the quantitative parameters, the mean, standard deviation and size will be calculated if they follow the Gauss Curve and if not (significant Shapiro Wilks test), the median and range will be calculated.
- In the time-dependent parameters, the Kaplan-Meier method will be applied, calculating the median in the time-to-event curve. In those cases where necessary, the 95% confidence interval will be calculated.

Main Objective Analysis

PFS (defined as time from start of treatment to progression or death from any cause) .

Secondary Objectives Analysis

• OS: Defined as the time from the start of treatment to death from any cause.

• Response Rate: Defined as the percentage of patients with Complete or Partial Response measured by RECIST criteria.

• S ecurity, be included all AEs that were reported during treatment with Trifluridine / Tipiracilo . AAs will be coded according to the MedDRA dictionary and will be presented classified by organs and systems (*System Organ Class* ; SOC) and preferred term.

• Efficacy relationship with GMI (Growing mass index)

• Relationship of the development of neutropenia with the efficacy variables.

Data management and quality control

All study data will be managed by a coordinating researcher.

6. ETHICAL ASPECTS

General ethical aspects

The study will be governed in accordance with the principles of the Declaration of Helsinki. Copies of the Declaration of Helsinki and subsequent amendments can be obtained through the website of the World Medical Association (http://www.wma.net/en/10home/index.html).

The study should be carried out in accordance with the protocol and standard work procedures that ensure compliance with the Good Clinical Practice (GCP) standards, as described in the ICH Tripartite Harmonized Standards for GCP 1996.

In accordance with international standards for conducting epidemiological studies, included in the *International Guidelines for Ethical Review of Epidemiological Studies* (*Council for the International Organizations of Medical Sciences*- CIOMS-Geneva, 1991) and the recommendations of the Spanish Society of Epidemiology Regarding the review of the ethical aspects of epidemiological research, this type of study must, except in certain specific cases, be subject to review by an accredited independent committee. Therefore, this study will be sent for approval to the accredited Clinical Research Ethics Committee (CEIC).

As it is a retrospective observational study, the data will be obtained from the clinical history of the patients. Their authorization will be requested after being informed about the nature and objectives of the study (Appendix 5).

Evaluation by the Ethics Committee for Clinical Research

Before the start of the study in any of the participating centers, the evaluation of the protocol by the CEICs of the participating centers will be required and the corresponding study approval report in writing will be necessary, in accordance with the current legislation applicable to observational studies of retrospective follow-up. The CEIC approval report will be filed with this document.

Modifications to the protocol

Any modification to the protocol, once approved by the CEIC, must be documented in writing, in the form of an amendment. The amendments will be duly identified, by their chronological order number, dated and signed by the promoter and the researcher. All amendments to the protocol will be notified to the CEICs involved in the trial prior to their application. If the modifications are relevant, the authorization of the CEICs involved is also necessary. The study investigators will be adequately informed of all modifications to the study protocol.

Benefit-risk assessment for participating subjects

Since it is an observational study, based on the retrospective review of medical records, the development of the study does not involve any risk for the patient.

Information Sheet and Informed Consent

In this study, patient information will be collected separately, in accordance with Organic Law 15/1999, of December 13, on the protection of personal data and Royal Decree 1720/2007, of December 21. Prior to data collection, it will be requested, as an essential prerequisite for the participation of patients in the study, the signing of the informed consent, by the patients or their legal representative, always before carrying out any activity related to the study.

The researcher, or a person designated by him, is responsible for obtaining informed consent, preferably in writing, or orally before independent witnesses of the research team, of each patient participating in this study, after having provided an adequate explanation of the purpose, methods. , objectives and possible risks of the study. It will not be necessary to obtain informed consent when this requires putting in place disproportionate means that make the study unviable, such as after the death of the patient.

In accordance with "Order SAS / 3470/2009, of December 16, which publishes the guidelines on observational post-authorization studies for medicinal products for human use", the information sheet for patients will be provided , attached with this protocol, so that they know the objective of the study, as well as that it is carried out without changing anything from their usual medical care, and that the data verification process will require access to some data from their medical history.

The signed informed consent will be filed in the researcher's file, so that they can be reviewed at any time during monitoring visits or during audits or inspections. A copy of the signed and dated document will be provided to the patient or their legal representative prior to their participation.

The ethics committee will be asked to exempt the signing of the informed consent in those patients already deceased at the beginning of the study.

Data confidentiality

Each patient will be assigned an identification code at the time of inclusion, immediately after signing the informed consent. This code will serve as the identifier of the patient in the study.

The patient data collected in the study will be stored under this identification code. The researcher will keep a separate record with the identification codes of the patients and their personal data such as full name, medical record number, which will allow him to keep track of the patients included in the study. Only the researcher may establish the relationship between said code and the patient's data, through the identification list that will remain confidential and will always remain in the researcher's file at the center.

The promoter and the researchers of the study must guarantee the confidentiality of the data of the subjects and ensure that the provisions of Organic Law 15/1999, of December 13, on the protection of personal data and the Royal Decree 1720/2007, of December 21.

The researcher must ensure that the confidentiality of the patient's data is maintained. In the study database or any other document sent to the promoter, patients cannot be identified by their name or other personal information, they can only be identified by their code. The researcher will conserve and maintain the confidentiality of documents not sent to the sponsor, such as the informed consent forms signed by the patient.

Interference with the doctor's prescribing habits

The present study is observational and retrospective review, therefore, there will be no interference with the doctor's prescription habits. The patients included in the study will be under medical care for their disease according to the doctor's usual clinical criteria and must only meet the inclusion and exclusion criteria detailed in the protocol.

7. MANAGEMENT AND NOTIFICATION OF ADVERSE EVENTS / ADVERSE REACTIONS

All AAs must register on the corresponding page (s) of the CRDe.

Security instructions

All events will be managed and documented in accordance with applicable regulations.

Definitions of Adverse Event (AA) and Serious Adverse Event (AAG)

An **Adverse Event** is any adverse medical circumstance that occurs in a patient or a patient in a clinical investigation who has been administered a pharmaceutical product, and that does not necessarily have to have a causal relationship with said treatment.

A **Serious Adverse Event** is any adverse medical circumstance that, at any dose:

- causes death, or
- is life threatening (the term "life threatening" within the definition of "severe" refers to an event in which the patient has suffered the risk of dying at the time of the event; it does not refer to an event that could have hypothetically caused death having been more serious)
- requires hospital admission or the extension of an existing hospitalization, or
- involves a significant or persistent disability / disability, or
- is a congenital anomaly / birth defect, or
- it is a medically important event. Overdose and pregnancy will be considered a major medical event,
- suspected transmission of an infectious agent; It is any suspicion of transmission of an infectious agent by a drug (for example , contamination of the product).

Medical and scientific judgment should be used to decide whether to proceed with expedited notification in other circumstances, such as important medical events that may not be immediately life threatening or lead to death or hospitalization but may harm the patient or require intervention to avoid any of the results collected in the previous definition.

Obligations of the Physician regarding the presentation of safety reports

Compilation of Adverse Events

All Adverse Events that occur from the signing of the informed consent to the end of the collection period as defined in the protocol for each patient, must be registered **immediately** (within 24 hours of being known) for the AGAs and in 30 days from when they are known for non-serious AA, on the corresponding page (s) of the Non-Data Collection Notebook or CRDe.

Whenever possible, the diagnosis or individual syndromes should be described rather than symptoms. The clinician should specify the onset date, intensity, Trifluridine / Tipiracil action taken, assigned corrective therapy / treatment, additional investigations conducted, results, and their opinion as to whether there is a reasonable possibility that AA is caused by Trifluridine / Tipiracil.

Laboratory, vital sign or ECG abnormalities will be recorded as AA only if they are medically relevant: symptomatic, leading to treatment modification (delay, reduction or interruption) or meeting a severity criterion .

Overdose

In the event of accidental or intentional symptomatic overdose (defined as the administration of any of the components of the combination at least 33% above the intended dose), even if it does not meet a criterion of severity, the representative of Laboratorios Servier (within 24 hours) by using the AA form that must be entered in the CRDe. However, overdoses without AA but requiring hospitalization are considered SAA (medically important event) and should be treated as such. In the event of an overdose, the patient should remain under observation for as long as the doctor deems appropriate and should be closely monitored. Appropriate symptomatic measures should be taken.

Pregnancy

Pregnancy occurring in a patient or spouse of a patient included in the study will be recorded as an AA in all cases. It will qualify as an AAG (Medically Important Event).

In Case of Non-Serious Adverse Events

All non-serious AAs must be entered in the corresponding screens of the CRDe within 30 days of becoming aware of the event.

Safety Observations

- The Physician must take all appropriate measures to ensure the safety of patients in accordance with normal practice.
- In the case of SAA, the patient should be followed until clinical recovery is complete and laboratory results have returned to normal, or until progression has stabilized. This may mean that follow-up continues until after the patient has left the study.

8. PLANS FOR THE DISSEMINATION OF THE RESULTS

All information related to the study is considered confidential and property of the promoter until its publication. The researcher agrees to keep this information confidential, and not to use it for any other purpose without the sponsor's written authorization.

Follow-up and final reports

Interim follow-up analysis / information is not foreseen

The definitive closure of the study will be carried out once all the data of the last patient included in the study have been completed. After closing the database, the statistical analysis will be carried out and a final report will be presented with the results, which will be reviewed and approved by the promoter of the study.

Once the study has finished and the statistical report has been made, the promoter, in collaboration with the researchers, will prepare the final report of the study, which will be sent to the AEMPS and the competent bodies of the Autonomous Communities where it was carried out. the same between three and six months after the end of the study. This final report will be the basis for the preparation of the manuscripts to be published in medical journals.

It is planned to present the results of the study in national and international conferences, as well as their publication in national and international scientific journals, with mention of the CEIC that approved the study, as well as the source of funding, guaranteeing at all times, the anonymity of the patients participating in it.

9. RESOURCES FOR CARRYING OUT THE STUDY AND ASSIGNING TASKS. FINANCING

The field work (review of medical records and data recording) will be carried out by the study investigators team, which will be made up of oncologists and health personnel from the participating centers.

The Researcher must complete all the required data in the excel form , following the guidelines described for completing the study , and transmit the data according to the procedures indicated by the promoter at the beginning of the study . Corrections in the forms will be made, if necessary, by the researchers themselves.

The management and analysis of the data will be carried out by a coordinating researcher.

The promoter will finance the expenses originated by the implementation and realization of the study, including the materials and logistics necessary for data capture, monitoring, and statistical analysis of the results. It is an observational study so it does not incur associated costs and in which no visits or laboratory analyzes will be made in addition to those necessary in normal clinical practice.

The sponsor and the researcher reserve the right to terminate the study at any time. If necessary, appropriate procedures will be prepared for each study after review, consultation and agreement by both parties. Once the study is finished, the sponsor and the researcher will ensure that the interests of the patients are adequately protected.

10. REFERENCES

1. Ferlay J, Soerjomataram I, Ervik M, Dikshit R, Eser S, Mathers C, Rebelo M, Parkin DM, Forman D, Bray, F. GLOBOCAN 2012 v1.0, Cancer Incidence and Mortality Worldwide: IARC CancerBase No. 11 [Internet]. Lyon, France: International Agency for Research on Cancer; 2013. Available from: http://globocan.iarc.fr, accessed on 12/13/2013

2. Ferlay J, Steliarova-Foucher E, Lortet-Tieulent J et al. Cancer incidence and mortality patterns in Europe: Estimates for 40 countries in 2012. Eur J Cancer 2013; 49: 1374-

1403]

3. Spanish Society of Medical Oncology (SEOM). Cancer figures in Spain 2017: Available at: https://www.seom.org/seomcms/images/stories/recursos/Las_cifras_del_cancer_en_Esp_2017.pdf

4. Cartwright TH. Treatment Decisions After Diagnosis of Metastatic Colorectal Cancer.

Clin Colorectal Cancer. 2012 Sep; 11 (3): 155-66.)

5. American Cancer Society. Colorectal Cancer Facts & Figures 2011-2013.

6. Venook A. Critical evaluation of current treatments in metastatic colorectal cancer. Oncologist. 2005; 10: 250-261.

7. Saltz LB, Cox JV, Blanke C, et al .: Irinotecan plus fluorouracil and leucovorin for metastatic colorectal cancer. Irinotecan Study Group. N Engl J Med 343 (13): 905-14, 2000.

8. De Gramont A, Figer A, Seymour M, et al .: Leucovorin and fluorouracil with or without oxaliplatin as first-line treatment in advanced colorectal cancer. J Clin Oncol. 2000; 18 (16): 2938-47.

9. Douillard JY, Cunningham D, Roth AD, et al .: Irinotecan combined with fluorouracil compared with fluorouracil alone as first-line treatment for metastatic colorectal cancer: a multicenter randomized trial. Lancet. 2000; 355 (9209): 1041-7.

10. Grothey A, Van Cutsem E, Sobrero A, Siena S, Falcone A, Ychou M, et al. Regorafenib monotherapy for previously treated metastatic colorectal cancer (CORRECT): an international, multicentre, randomized, placebo-controlled, phase 3 trial. Lancet. 2013; 381: 303–312.

11. Mayer RJ, Van Cutsem E, Falcone A, Yoshino T, Garcia-Carbonero R, Mizunuma N, RECOURSE Study Group, et al. Randomized trial of TAS-102 for refractory metastatic colorectal cancer. N Engl J Med. 2015; 372: 1909–1919.

12. Van Cutsem E, Cervantes A, Nordlinger B, Arnold D, On Behalf of the ESMO Guidelines Working Group. Metastatic colorectal cancer: ESMO Clinical Practice Guidelines for diagnosis, treatment and follow-up Decision making. Ann Oncol. 2014; 25 (suppl. 3): iii1–9.

13. Casado-Saenz E, Feliu J, Gomez-Espan˜a MA, Sanchez-Gastaldo A, Garcia- Carbonero R. SEOM clinical guidelines for the treatment of advanced colorectal cancer 2013. Clin Transl Oncol. 2013; 15: 996–1003.

14. Tanaka N, Sakamoto K, Okabe H, et al. Repeated oral dosing of TAS-102 confers high trifluridine incorporation into DNA and sustained antitumor activity in mouse models. Oncol Rep 2014; 32: 2319-2326.

15. Fukushima M, Suzuki N, Emura T, et al. Structure and activity of specific inhibitors of thymidine phosphorylase to potentiate the function of antitumor 2′-deoxyribonucleosides. Biochem Pharmacol 2000; 59: 1227-1236.

16. Emura T, Murakami Y, Nakagawa F, Fukushima M, Kitazato K. A novel antimetabolite, TAS-102 retains its effect on FU-related resistant cancer cells. Int J Mol Med 2004; 13: 545-549.

17. Emura T, Suzuki N, Fujioka A, Ohshimo H, Fukushima M. A novel combination antimetabolite, TAS-102, exhibits antitumor activity in FU-resistant human cancer cells through a mechanism involving FTD incorporation in DNA. Int J Oncol 2004; 25: 571-578.

18. Hong DS, Abbruzzese JL, Bogaard K, et al. Phase I study to determine the safety and pharmacokinetics of oral administration of TAS-102 in patients with solid tumors. Cancer 2006; 107: 1383-1390.

19. Overman MJ, Varadhachary G, Kopetz S, et al. Phase 1 study of TAS-102 administered once daily on a 5-day-per-week schedule in patients with solid tumors. Invest New Drugs 2008; 26: 445-454.

20. Overman MJ, Kopetz S, Varadhachary G, et al. Phase I clinical study of three times a day oral administration of TAS-102 in patients with solid tumors. Cancer Invest 2008; 26: 794-799.

21. Green MC, Pusztai L, Theriault LR, et al. Phase I study to determine the safety of oral administration of TAS-102 on a twice daily (BID) schedule for five days a week (wk) followed by two days rest for two wks, every (Q) four wks in patients (pts) with metastatic breast cancer (MBC). Proc Am Soc Clin Oncol 2006; 24: 10576.

22. Patel MR, Bendell JC, Mayer RJ, et. to the. A phase I dose-escalation study of TAS-102in patients (pts) with refractory metastatic colorectal cancer (mCRC). J Clin Oncol 2012; 30: 3631. Abstract.

23. Yoshino T, Mizunuma N, Yamazaki K, et al. TAS-102 monotherapy for pretreated metastatic colorectal cancer: a double blind, randomized, placebo-controlled phase 2 trial. Lancet Oncol 2012; 13: 993-1001.

23. Yoshino T, Mizunuma N, Yamazaki K, et al. TAS-102 monotherapy for pretreated metastatic colorectal cancer: a double-blind, randomized, placebo-controlled phase 2 trial. Lancet Oncol 2012; 13: 993-1001.

11. APPENDICES

APPENDIX 1. DATA COLLECTION NOTEBOOK

An electronic CRD will be used.
